# Mid-Term and Long-Lasting Psycho–Cognitive Benefits of Bidomain Training Intervention in Elderly Individuals with Mild Cognitive Impairment

**DOI:** 10.3390/ejihpe14020019

**Published:** 2024-01-26

**Authors:** Ines Ben Ayed, Chirine Aouichaoui, Achraf Ammar, Salma Naija, Oussama Tabka, Haitham Jahrami, Khaled Trabelsi, Yassine Trabelsi, Nicole El Massioui, Farid El Massioui

**Affiliations:** 1 Research Laboratory, Exercise Physiology and Physiopathology: From Integrated to Molecular “Biology, Medicine and Health”, LR19ES09, Faculty of Medicine of Sousse, Sousse University, Sousse 4000, Tunisia; ines.benayed@isseps.usf.tn (I.B.A.); chirineaouichaoui@yahoo.com (C.A.); otabka@ghpsj.fr (O.T.); yassine.trabelsi@issep.uma.tn (Y.T.); 2 Laboratory of Human and Artificial Cognition (EA 4004), Psychology UFR, University of Vincennes/Saint-Denis, 93200 Saint-Denis, France; nelmassioui@gmail.com; 3 Research Laboratory, Education, Motricity, Sport and Health (EM2S), LR15JS01, High Institute of Sport and Physical Education of Sfax, University of Sfax, Sfax 3000, Tunisia; trabelsikhaled@gmail.com; 4 High Institute of Sport and Physical Education of Ksar Saïd, University of Manouba, Mannouba 2010, Tunisia; 5 Department of Training and Movement Science, Institute of Sport Science, Johannes-Gutenberg-University Mainz, 55122 Mainz, Germany; 6 Research Laboratory, Molecular Bases of Human Pathology, LR19ES13, Faculty of Medicine of Sfax, University of Sfax, Sfax 3000, Tunisia; 7 Interdisciplinary Laboratory in Neurosciences, Physiology and Psychology: Physical Activity, Health and Learning (LINP2), UFR STAPS, UPL, Paris Nanterre University, 92000 Nanterre, France; 8 High Institute of Sport and Physical Education of Sfax, University of Sfax, Sfax 3029, Tunisia; 9 Neurology Department, University Hospital Sahloul Sousse, Sousse 4052, Tunisia; naijasalma@gmail.com; 10 College of Medicine and Medical Science, Arabian Gulf University, Manama 293, Bahrain; hjahrami@health.gov.bh

**Keywords:** MCI, aging, cognitive decline, physical activity, low-cost intervention, combined intervention, problem-solving, working memory, selective attention

## Abstract

*Background:* This study investigated whether combining simultaneous physical and cognitive training yields superior cognitive outcomes compared with aerobic training alone in individuals with mild cognitive impairment (MCI) and whether these benefits persist after four weeks of detraining. *Methods:* Forty-four people with MCI (11 males and 33 females) aged 65 to 75 years were randomly assigned to an 8-week, twice-weekly program of either aerobic training (AT group, *n* = 15), aerobic training combined with cognitive games (ACT group, *n* = 15), or simply reading for controls (CG group, *n* = 14). Selective attention (Stroop), problem-solving (Hanoi Tower), and working memory (Digit Span) tasks were used to assess cognitive performances at baseline, in the 4th (W4) and 8th weeks (W8) of training, and after 4 weeks of rest (W12). *Results:* Both training interventions induced beneficial effects on all tested cognitive performance at W4 (except for the number of moves in the Hanoi tower task) and W8 (all *p* <0.001), with the ACT group exhibiting a more pronounced positive impact than the AT group (*p* < 0.05). This advantage was specifically observed at W8 in tasks such as the Stroop and Tower of Hanoi (% gain ≈40% vs. ≈30% for ACT and AT, respectively) and the digit span test (% gain ≈13% vs. ≈10% for ACT and AT, respectively). These cognitive improvements in both groups, with the greater ones in ACT, persisted even after four weeks of detraining, as evidenced by the absence of a significant difference between W8 and W12 (*p* > 0.05). Concerning neuropsychological assessments, comparable beneficial effects were recorded following both training regimens (all *p* < 0.05 from pre- to post-intervention). The control group did not show any significant improvement in most of the cognitive tasks. *Conclusions:* The greater mid-term and long-lasting effects of combined simultaneous physical–cognitive training underscores its potential as a cost-effective intervention for the prevention and management of cognitive decline. While these results are valuable in guiding optimal physical and mental activity recommendations for adults with MCI, further neurophysiological-based studies are essential to offer robust support and deepen our understanding of the mechanisms underlying these promising findings.

## 1. Introduction

Regarding the projected doubling of the global population aged over 60 by 2050 (reaching 2 billion) and considering the alarming annual incidence of 7.7 million new cases of dementia (projected to be 75.6 million in 2030 and 135.5 million in 2050 [1]), there is an urgent need to define optimal low-cost intervention to mitigate the rise of new dementia cases. In particular, specific attention should be directed toward older adults already experiencing cognitive decline, such as mild cognitive impairment (MCI) [2]. MCI is the intermediate stage between normal age-related cognitive decline and dementia [3]. Adults with MCI exhibit cognitive deficits, including impaired memory, attention, orientation, and executive functions [4]. With an annual conversion rate of 10–30% of individuals with MCI progressing to Alzheimer’s disease (AD), as opposed to the 1–2% annual rate in healthy adults of the same age [4,5]. Therefore, given the current absence of drug therapy and the increasing prevalence of dementia [6], nonpharmacological interventions are necessary.

Chronic exercise intervention has demonstrated a beneficial effect on preventing cognitive impairment in older adults [7,8]. It is also effective in preventing chronic pathologies (e.g., diabetes, cancer, chronic obstructive pulmonary disease, and depression) [9] and appears to be a powerful tool for delaying neurodegenerative processes [10]. The improvement in cognitive performance can be attributed to the enhancement of global cerebral functional connectivity [11] and, particularly, the facilitation of prefrontal cortex activation [12,13]. In adults with MCI, aerobic training has been shown to improve global cognition, including logical memory, inhibitory control, divided attention, conflict resolution, processing speed, and verbal fluency [14,15]. Compared with medication (e.g., cholinesterase inhibitors and memantine) [16,17], physical exercise interventions demonstrate fewer side effects and better adherence [18].

Although physical exercise has demonstrated positive effects on the cognition of adults with MCI and dementia patients, providing specific practical recommendations, such as the type, frequency, intensity, or duration of exercise, remains challenging for these populations [19]. Nevertheless, it is crucial to note that the regularity and continuity of exercise training play a pivotal role in improving cardiovascular health and cognitive performance in MCI subjects [2,19,20]. A previous report indicated that even a four-week training cessation can generate a loss of training-induced beneficial effects in individuals with MCI [2]. Importantly, incorporating cognitive tasks during exercise has been suggested to enhance these positive effects [21].

Combining a single physical training intervention with a single cognitive training (referred to as a combined training) would greatly increase the likelihood of cognitive benefit. The time course of these training-induced effects is still a subject of debate. Studies have shown that processing speed and executive function displayed benefits after only 3 weeks of training, whereas memory improvement needed 12 weeks [22,23]. Conversely, following the cessation of training, therapeutic gains for memory benefits decay quickly, while speed effects remain durable for at least 3 months. However, a different study in older adults with subjective memory impairments [24] found that a 4-week simultaneous memory training and aerobic exercise program was sufficient to improve composite/overall memory functioning. Moreover, meta-analysis by Zhu et al. [25] revealed a modest impact of these combined interventions, both sequential and simultaneous, on attention, executive function, visuospatial ability, memory, and global cognition when compared with a control group (*p* < 0.001 and effect size (ES) = 0.29) or exercise training alone (*p* < 0.01 and ES = 0.22). However, there were no significant differences between the effects of combined interventions and cognitive training alone. Secondary analysis indicated that the ES for sequential (ES = 0.27) interventions was lower than that for simultaneous (ES = 0.43) interventions in healthy older adults. The current result is comparable with a published meta-analysis by Karssemeijer et al. [26], who reported that combined cognitive and physical exercise interventions are beneficial for activities in daily living and mood in older adults with MCI or dementia, emphasizing the clinical relevance of combined cognitive and physical exercise training strategies. However, some studies did not observe the cognitive benefits of the combined intervention on global cognition in older adults with MCI [27,28], while a new study has shown its positive effect [29]. It is urgent to update the data.

To explore the effectiveness of the combined interventions, the present study aims to evaluate whether elderly individuals with MCI would experience greater benefits, in terms of cognitive and aerobic performances, from combining cognitive training with aerobic cycling exercise compared with aerobic training alone. Additionally, the study explores whether such benefits can persist following four weeks of detraining. Our research team recently reported the beneficial acute effects of similar combined exercises in Alzheimer’s disease patients after a single session [30]. Therefore, we hypothesized that, compared with aerobic-based training, 8 weeks of combined training would induce greater mid-term and long-lasting effects on cognitive performance in individuals with MCI.

## 2. Materials and Methods

### 2.1. Design of the Study

**Figure 1:**
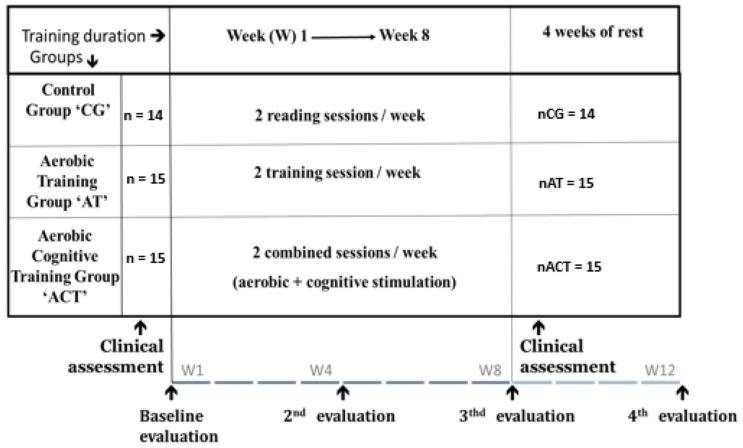
Experimental design.

An 8-week randomized controlled trial of training interventions was carried out at the Laboratory of Exercise Physiology and Physiopathology (LR19ES09) in the Faculty of Medicine of Sousse, Tunisia. Cognitive assessments were conducted at baseline, midpoint, at the end of the training, and after a 1-month follow-up (4 weeks of detraining). The cognitive evaluation was randomized and counterbalanced for all adults with MCI. These evaluations were conducted under the supervision of a PhD student. For the cognitive assessments of elderly participants in this study, a comprehensive neuropsychological evaluation was administered by a qualified neuropsychologist. Neuropsychological assessments were administered to all participants at baseline and at the end of the training (see Figure 1).

### 2.2. Participants

The minimum required sample size was calculated a priori based on procedures suggested by Beck [31] and using the software G∗power (version 3.1.9.2; Kiel University, Kiel, Germany) [32]. The F test family (ANOVA: repeated measures, within-between interaction) with three groups and four measurements was used. The probability of type I (α ≤ 0.05) error was fixed at 0.05, and the value of power (1-β error probability) was fixed at 0.9. The assumed correlation between the repeated measures was 0.5. Based on the study of Ben Ayed et al. [30] and discussions between the authors, the effect size as partial eta-squared was estimated to be 0.06 (medium effect), corresponding to an effect size (f) of 0.25. The minimum required sample size for this study was 39.

A total of 44 adults with MCI voluntarily participated in the study. All 44 individuals with MCI successfully completed the intervention over an 8-week period, followed by 4 weeks of rest. Study participants were clinically diagnosed with MCI according to the International Working Group diagnostic criteria (IGW2) [33] at the neurology department of Sahloul Hospital in Sousse, Tunisia.

The inclusion criteria for study participation were as follows: aged between 65 and 75 years, living independently in a noninstitutional environment, scoring ≥ 26/30 on the Mini Mental State Examination (MMSE) [34], having normal or corrected-to-normal vision and color perception, and being able to move on a daily basis without technical assistance. Exclusion criteria included the use of medication affecting exercise capacity or posture, regular participation in aerobic training for the last six months, cardiovascular or other diseases that do not allow physical activity, other neurological diseases that may provoke cognitive decline (including severe cerebrovascular diseases detected after cranial computed tomography or magnetic resonance imaging), and a diagnosed severe psychiatric disease (e.g., depression).

The experimental protocol was approved by the Research Ethics Committee of the Faculty of Medicine of Sousse, Tunisia (IRB 00008931).

All participants provided written, informed consent. Adults with MCI were randomly assigned to an aerobic training group (AT), an aerobic-cognitive training group (ACT), or a control group (CG).

### 2.3. Interventions

The AT consisted of a twice-weekly pedaling exercise for 8 weeks. Before the exercise, the seat on the bicycle ergometer (Labyrinth, Paris, France) was adjusted to the participant’s height. Immediately after a 5-min warm-up, the participant performed a 20-min session of exercise at a moderate intensity (i.e., 60% of the maximal heart rate) [35,36], followed by a 5- to 10-minute cool-down. Maximum heart rates were measured during a familiarization session at the end of the 6 min walk test (6MWT), one week prior to the experimentation period [35,36]. Participants were monitored using heart rate monitors (Polar V800, USA) during the exercise sessions, and they were asked to report their rates of perceived exertion at the end of each session.

According to the results of meta-analysis by Zhu et al. [25], the ACT combined training involved simultaneous physical exercise and cognitive training in a dual-task format. Participants in this group followed the same training program as the AT group, with the additional inclusion of three easy cognitive games displayed on a computer screen. This cognitive training consisted of simple and recreational cognitive exercises involving, within the same session, different cognitive processes such as information processing speed, selective attention, working memory, arithmetic, scanning, visual, or even temporal perception. These games were developed by a team of neuropsychologists and medical specialists to train cognitive activity in elderly MCI subjects. Each game lasted approximately 5 min. To prevent familiarization, three different games from the previous session were presented at each training session. The scores for these cognitive tasks were not measured, as they were designed to complement the physical exercise.

Adults with MCI in the CG were instructed to read for 20 min instead of engaging in pedaling exercises.

### 2.4. Measures

Neuropsychological assessments were employed for subjective assessments of the ability to perform daily activities, including the MMSE test (the cutoff values for screening MCI are 26/27) [34,37], the 16-item free and cued recall task (RL/RI-16) [38] (the scoring criteria consider the performance to be in the pathological range if the patient provides fewer than 10 responses for phonemic fluency and 15 responses for categorical fluency), the clock task scored on a scale of 7 points (normal score) [39], and the fluency test [40]. Additionally, the 15-item Geriatric Depression Scale (GDS) (the optimal cut-off score is 7) [41] was utilized for depression screening, as was the World Health Organization Quality of Life-BREF (WHOQOL-BREF-100) [42]. This self-report questionnaire consists of 26 items that measure four domains, including physical and psychological health and social and environmental relationships. According to the WHO, the quality-of-life thresholds are interpreted for each of the abovementioned domains as follows: poor (6–16, 6–14, 3–7, and 8–18, respectively), average (17–26, 15–22, 8–11, and 19–29, respectively), and good (27–35, 23–13, 12–15, and 30–40, respectively). The total score ranges from 26–60 for poor quality of life, 61–95 for average quality of life, and 96–130 for good quality of life.

Cognitive functions (i.e., selective attention, working memory, and problem-solving) were evaluated 5 to 10 min after the end of the exercises to allow the heart rate to return to a resting status (10% above the individual baseline rate). Cognitive evaluations were randomly counterbalanced across all individuals with MCI to avoid an order effect. The entire experiment was conducted by the same person. After giving instructions, the experimenter sat next to the subjects and followed their performance without any reaction.

Moreover, these tasks were treated as experimental situations and not as tests with pre-established standards. A comparison of the performance between participants in the experimental groups and those in the control group indirectly validated the hypothesis in question.

The Stroop color word test (French version of Victoria) assessed selective attention [43] and resistance to interference [44]. In the present study, an experimental condition was added: reading color names written in black. The objective of this additional assessment (see Ben Ayed et al. [30] for procedural details) was to examine automatic reading behavior with a small number of items to create the conditions for interference.

The digit span task was used to measure working memory [45,46]. In the middle of the computer screen, a series of digits appeared at a rate of one digit per second. In two experimental conditions, subjects had to recall a series of digits, either forward or backward. Performance was measured by the number of recalled items. The cut-off points for the Digit Forward and Backward scores were set at 4 [47], and the complete procedure was described in detail in our previous study [30].

Problem-solving skills were explored using the Tower of Hanoi test [48]. Participants were needed to rebuild a three-disk pyramid from the right-hand peg to the left peg, using the middle one for necessary moves. Subjects must move only one disc at a time and never place a large disk on a smaller disc. The number of moves and times taken were measured. The minimum number of moves to solve the three-disk Tower of Hanoi is seven.

### 2.5. Statistical Analysis

Analyses were conducted using SPSS software (IBM^®^ SPSS^®^ Statistics version 26). Descriptive statistics, including the mean (M) and standard deviation (SD) for the MMSE, Stroop, Tower of Hanoi, and digit span tests, were calculated. Additionally, to estimate the magnitude of significant differences between the baseline and post intervention values (W4, W8, and W12), the percent of performance gain was calculated as follows: [(post value−pre value)/(pre value)] × 100. The normality of the distribution was checked using the Shapiro–Wilks W-test.

The Kruskal–Wallis nonparametric test was used to explore the main effect of groups. When appropriate, post hoc pairwise comparisons were performed, and the results were interpreted using a Bonferroni correction. The Friedman test was employed to explore the time assessment (baseline, W4, and W8) of each group. Within-group results of neuropsychological measures (Baseline and W8) and the persistent effect (W8 and W12) were interpreted according to the paired Wilcoxon test. In our study, the dependent variables were cognitive performance (i.e., Tower of Hanoi, Stroop, and digit span tests), whereas age, disease duration, education level, sex, and group served as independent variables. Significance of all analyses was accepted at the level of *p* < 0.05.

## 3. Results

### 3.1. Participant Characteristics

Forty-four diagnosed adults with MCI were randomly included in the three groups. Neuropsychological data assessments confirmed that all participants constituted a homogenous sample, as no significant differences were found between the groups (*p* > 0.05) in any of the tested parameters (see Table 1).

### 3.2. Neuropsychological Results

After the 8-week intervention program, participants in the ACT and AT groups showed significant improvements in all neuropsychological scores (all *p* < 0.05; detailed statistics are in Table 2). The CG participants exhibited a significant difference (pre- and post-intervention) only in GDS scores (*p* = 0.000). See detailed statistics in Table 2.

### 3.3. Training and Detraining Effects on Cognitive Performance

**Figure 2:**
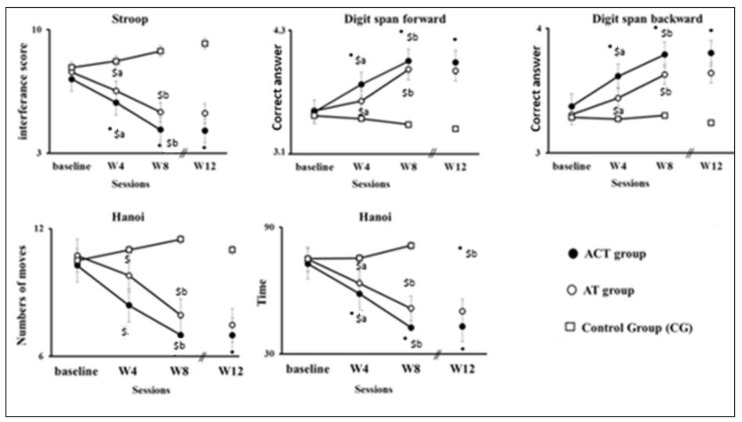
Performances in the different tasks for groups CG, AT, and ACT before training (baseline), after 4 (W4) or 8 weeks (W8) of training, and after 1 month of rest (W12). *: significant difference between AT and ACT; $: significant differences compared with CG; a: significant difference between baseline and W4; and b: significant differences between W4 and W8.

As shown in Figure 2 (detailed results presented in Table S1), regardless of the cognitive task, there were no differences between groups at baseline. After four weeks of training (W4), both the AT and ACT groups significantly improved the majority of their cognitive performances (except Hanoi moves) compared with the CG group (all *p* < 0.001), with ACT achieving greater digit span backward performance than AT (*p* = 0.000). The performance of the AT and ACT groups continued to improve at W8 compared with W4 (all *p* < 0.01; see detailed statistics in Table S1), with ACT outperforming AT in all tested skills (all *p* < 0.05). It is worth noting that the CG group’s performance remained unchanged throughout the 8-week intervention program. Following 4 weeks of detraining, a lasting effect was observed in the AT and ACT groups in all tested tasks (Figure 2), as evidenced by the absence of a significant difference between W8 and W12 (detailed results presented in Table S2). The improvements induced by training at W8 persisted at W12.

**Figure 3:**
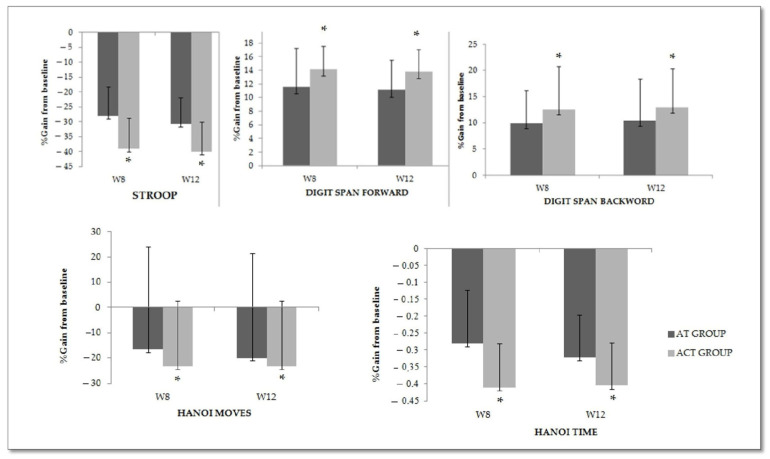
Percentage gain from baseline at W8 and W12 for the different tasks of the AT and ACT groups. *: significant difference between AT and ACT.

Figure 3 presents the percentage gain in cognitive performance registered at W8 and W12 compared with the baseline values. Overall, higher percentages of gain were recorded for ACT group compared with AT group in all tested tasks at both W8 and W12. These differences were statistically significant for the Stroop and Hanoi time tests (i.e., percent gains: ≈40% vs. ≈30% for ACT and AT, respectively) at both test sessions (all *p* < 0.05), as well as for the digit span forward at W12 (*p* = 0.04) and percent gains: ≈13% vs. ≈10% for ACT and AT, respectively).

## 4. Discussion

The aims of this study were to investigate whether eight weeks of combining simultaneous physical and cognitive training yields superior cognitive outcomes compared with aerobic training alone in individuals with MCI and whether these benefits persist after four weeks of detraining. The main findings revealed that both training protocols induced beneficial effects on cognitive performance, specifically in tasks such as the Stroop, Digit Span, and Tower of Hanoi (completion time) tasks, with the combined training exhibiting a more pronounced positive impact compared with both the aerobic training and control groups. Notably, the cognitive improvements observed in the combined and aerobic groups persisted even after four weeks of detraining, suggesting a lasting effect. These results highlight the advantage of simultaneously incorporating game-based cognitive tasks into the physical exercise training intervention, yielding greater mid-term (at 4 and 8 weeks of training) and long-lasting (after 4 weeks of detraining) effects on executive functions in adults with MCI. Concerning neuropsychological assessments, comparable beneficial effects were recorded following both training regimens.

Looking at an optimal training protocol characterized by inducing beneficial effects within a short engagement duration, the present study showed that engaging in only two sessions a week of aerobic exercises for an 8-week period was sufficient to induce a significant improvement in executive functions in individuals with MCI. This contrasts with earlier research, which often employed longer interventions to achieve similar effects [49]. A positive effect on memory was previously found after six-month training using either physical activities or memory rehabilitation exercises [50]. Similarly, cognitive improvements have been observed following an extended period of physical exercise (24 weeks) [51,52]. Importantly, our key findings suggest that when simultaneously combining aerobic-based training with game-based cognitive training, which is supposed to be less sensitive to repetition than classical neuropsychological tools, more pronounced advantages are registered. For instance, in the Stroop test, we observed a reduction in interference sensitivity after aerobic exercises alone (AT group), with a gain of approximately 30%. This improvement was even more pronounced when aerobic exercises were combined with cognitive tasks (ACT group), resulting in a gain of approximately 40%. In the Tower of Hanoi task, adults with MCI demonstrated increased speed and accuracy after cycling, and this improvement was further enhanced when cycling was combined with cognitive games. Similarly, the assessment of working memory using the digit span task revealed a positive effect of aerobic exercises, which further improved when combined with cognitive activities compared with the control group. Interestingly, our study aligns with previous research that highlighted the potential superiority of combined interventions over single physical or cognitive interventions [10,53]. A recent meta-analysis study by Meng et al. [53] reported that combined physical and cognitive interventions demonstrated superiority over single physical exercise or single cognitive intervention on memory and executive function (e.g., attention). Similarly, Gavelin et al. [54] suggested that simultaneously combined interventions are efficacious for promoting cognitive and physical health in older adults as well as for people with MCI and therefore should be preferred over the implementation of single-domain training. The current result is comparable with a published study by Salzman et al. [55], indicating that nonpharmacological, multidomain interventions primarily emphasized areas such as physical and cognitive training, dietary supplements, music, mind-body, education, and social engagement. These interventions have demonstrated small to medium ES-revealing enhancements in global cognition, memory, executive function, and verbal fluency. Additionally, a synergistic relationship has been identified, suggesting that combined interventions may offer superior benefits compared with single interventions for enhancing cognitive functioning in older adults with MCI. Recently, the results of pairwise meta-analyses by Xue et al. [56] indicated that combined interventions were superior to exercise in improving working memory, delayed recall, depression, and activities of daily living in the MCI population. Concerning quality of life measures, comparable beneficial effects were recorded following both training regimens. In line with this, the trial results of Uysal et al. [57] proved that aerobic exercise and dual-task training are the best combination for improving cognitive status, mood, and quality of life in older adults with MCI.

The observed cognitive benefits from the eight-week combined training program are likely attributable to the enhancement of the neural and vascular mechanisms, facilitated through the promotion of neurogenesis, brain angiogenesis, and synaptic plasticity [58,59]. Ten Brinke et al. [52] demonstrated a significant increase in hippocampal volume in older women with probable MCI following 6 months of aerobic training, which was independently associated with improved verbal memory and learning performance. This finding underscores the potential of exercise-induced structural changes in the brain to positively influence cognitive outcomes. Furthermore, after 12 weeks of moderate-intensity walking exercise training, Chirles and colleagues [60] observed increased functional connectivity of the posterior cingulate cortex (PCC)/pecuneus. Given that this region is preferentially affected in Alzheimer’s pathology and in individuals with MCI, the heightened connectivity may indicate an augmented cognitive reserve [60,61]. Systematic reviews indicate that both physical [62] and cognitive training [63] lead to positive neurobiological changes with potential therapeutic relevance. The therapeutic premise of combined interventions is that physical and cognitive interventions might influence brain plasticity through distinct and complementary pathways, whereby physical exercise induces physiological changes (e.g., upregulation of brain-derived neurotrophic factor (BDNF) and stimulation of hippocampal neurogenesis). Alterations in BDNF levels may occur early in the course of MCI and AD and may contribute to their progression [64]. In older adults, both mental [65,66] and physical [67,68] activities have been shown to significantly elevate BDNF, with physical activity being particularly effective in immediately increasing BDNF serum levels compared with cognitive training and meditation [69]. This highlights the distinct mechanism by which physical activity enhances brain function. Improvement in cognitive functions also appears to be substantially linked to enhanced brain oxygenation [70], facilitated by increased cerebral blood flow (CBF) during exercise for healthy individuals [71]. Considering that a decrease in CBF occurs in individuals with MCI prior to the transition to Alzheimer’s disease [72], engaging in aerobic exercise training may act as a preventive or decelerating measure against pathological cognitive decline by increasing CBF, as suggested by Park et al. [51]. Accordingly, it is speculated that the current combination of aerobic-based exercise and cognitive training stimulates various mechanisms underlying cerebral functioning, potentially contributing to a significant mid-term and long-lasting improvement in executive functions in individuals with MCI. However, future neurophysiological-based studies are needed to test this speculation and precisely identify the involved mechanisms.

Importantly, even after four weeks of training cessation, the greater gain obtained on executive functions following the 8-week combined training persisted in our MCI participants, showing no significant difference compared with the performance at W8. These findings underscore the long-lasting effect of the physical–cognitive training combination, presenting a promising avenue in the field of rehabilitation. When considering the emotional well-being of the participants, positive effects have been observed on quality of life and depression scores, regardless of the enrollment group. It is worth noting that, after only 8 weeks of training, the depression scores of our experimental groups reached the standard level of healthy subjects. While promising, the present results should be interpreted with caution due to the small sample size.

## 5. Strengths and Limitations

The present study presents a pioneering effort in assessing the effects of an 8-week combined physical–cognitive training program on the cognitive functions of elderly individuals already experiencing cognitive decline while controlling retention performance following 4 weeks of detraining. The strength of this study lies in its approach, which seeks to identify an optimal intervention protocol capable of inducing a beneficial impact on the tested parameters and the target population, all within a comparatively shorter engagement duration than previously recommended protocols. The most common duration of previous study interventions ranges between six and twelve weeks [9]. However, it is recognized that cognitive improvements may manifest over an extended period, with certain domains exhibiting progress later than others [9]. Remarkably, our study demonstrated positive effects within a relatively short training duration. Additionally, we opted for cycling exercises, given their documented cognitive benefits compared with walking or running exercises [73]. Notably, our research is distinctive in its clinical focus, aiming to elucidate the potential for maximizing cognitive benefits through combined training compared with physical training alone, which is considered a leading nonpharmacological therapy [74]. The group engaging in training simultaneously with cognitive games exhibited a notably superior effect, and this positive impact persisted for at least four weeks post-training.

The primary limitations of our study were the small size of the sample, which was influenced by various factors, including stringent inclusion and exclusion criteria. Additionally, the absence of neurophysiological measurements (e.g., CBF, BDNF, EEG, fNIRS, etc.) represents another limitation. These measurements could have provided valuable insights into the underlying mechanisms that contribute to the promising findings observed in our study. Furthermore, the absence of measurements related to exercise and cognitive habits during the detraining period represents another limitation of our study. Future research incorporating a larger and more diverse sample size, along with comprehensive neurophysiological assessments, is warranted to further validate and elucidate the mechanisms behind the observed effects.

## 6. Conclusions and Future Recommendations

With increasing life expectancy and demographic aging, the global prevalence of Alzheimer’s disease is anticipated to rise. Consequently, it is imperative to urgently explore solutions aimed at slowing down the emergence of new dementia cases. The present study offers promising findings that endorse simultaneously combined physical–cognitive training as a potential candidate for cost-effective intervention for the prevention and management of cognitive decline and, thereby, neurodegenerative diseases. More research is needed to determine whether cognitive and physical improvements following combined interventions translate into enhanced everyday function and well-being. This is particularly crucial for individuals with cognitive and functional impairments. Additionally, investigations are needed to identify the specific components that constitute an effective combined intervention and to establish the regimens needed for maintaining long-term gains. While these findings hold significant practical implications and may open new research avenues in the prevention and therapeutic domains, it is essential to underscore that further neurophysiological-based studies are needed to provide robust support and deepen our understanding of these results.

## Figures and Tables

**Table 1:** Baseline participant characteristics (*n* = 44).

Variable	CG (*n* = 14)	AT (*n* = 15)	ACT (*n* = 15)
	M (SD)	M (SD)	M (SD)
Age (years)	69.24 (4.84)	67.93 (5.18)	67.13 (3.04)
Age of the onset of the disease (years)	64.57 (4.44)	63.26 (4.75)	62.00 (3.83)
Height (cm)	160.35 (5.66)	170.00 (10.59)	163.60 (8.34)
Weight (kg)	73.71 (8.35)	82.20 (9.03)	79.06 (10.63)
Education level	5.00 (0.65)	4.00 (1.00)	4.20 (0.94)
Sex (M/F)	2/12	5/10	5/10
MMSE (30 point max)	26.07 (0.26)	26.20 (0.56)	26.13 (0.35)
GDS (15 point max)	6.21 (0.57)	5.93 (0.45)	5.20 (0.41)
WHOQOL-BREF-100 (100 point max)	76.91 (6.06)	77.92 (5.68)	78.65 (7.13)
Six-Minute Walk Test (m)	470 (57.13)	479 (56.10)	473 (58.39)

M = Mean; SD = Standard Deviation; F = female; M = male; MMSE = Mini-Mental Status Examination; GDS = Geriatric Depression Scale; WHOQOL-BREF = World Health Organization Quality of Life-BREF.

**Table 2:** Neuropsychological measures of the three groups (CG, AT, and ACT) before and after activities (reading, pedaling, and combined).

	CG (*n* = 14)			AT (*n* = 15)			ACT (*n* = 15)		
	Baseline	W8		Baseline	W8		Baseline	W8	
	M (SD)	M (SD)	Z	M (SD)	M (SD)	Z	M (SD)	M (SD)	Z
MMSE	26.07 (0.26)	26.07 (0.26)	0.00	26.20 (0.56)	26.93 (0.79)	−2.49 *	26.13 (0.35)	27.13 (0.83)	−2.74 **
RL/RI-16	13.14 (1.16)	13.37 (1.08)	−1.00	13.13 (1.18)	14.06 (0.79)	−2.55 *	13.00 (1.25)	14.80 (0.86)	−3.17 ***
Letter fluency	10.71 (0.72)	10.57 (0.51)	−1.41	10.53 (0.74)	11.53 (1.35)	−2.72 **	10.80 (0.77)	12.33 (0.89)	−3.38 ***
Category fluency	13.07 (0.82)	13.07 (0.82)	0.00	13.86 (0.99)	14.33 (0.61)	−2.07 *	13.33 (0.97)	14.80 (0.86)	−3.10 **
Clock test	5.57 (0.64)	5.71 (0.61)	−1.41	6.00 (0.53)	6.46 (0.51)	−2.33 *	5.66 (0.48)	6.53 (0.51)	−2.92 **
GDS 15 items	6.21 (0.57)	5.07 (0.61)	−3.08 ***	5.93 (0.45)	4.60 (0.63)	−3.54 ***	5.20 (0.41)	3.80 (0.67)	−3.25 ***
WHOQOL-BREF-100 D1	21.71 (1.43)	21.92 (1.32)	−1.73	21.00 (1.36)	22.46 (1.84)	−2.84 **	21.73 (1.83)	23.40 (2.03)	−2.83 **
WHOQOL-BR EF-100 D2	19.35 (1.78)	19.35 (1.78)	0.00	20.86 (1.30)	22.06 (1.22)	−2.84 **	20.33 (1.54)	22.60 (1.72)	−3.21 ***
WHOQOL-BREF-100 D3	09.64 (0.74)	08.79 (1.52)	−1.56	9. 06 (1.38)	8.06 (0.88)	−2.87 **	9.67 (1.40)	10.47 (1.55)	−2.76 **
WHOQOL-BREF-100 D4	26.21 (2.11)	24.93 (1.54)	−2.20	27.00 (1.64)	27.96 (1.50)	−2.23 *	26.93 (2.37)	28.80 (1.66)	−2.68 **

MMSE = Mini-Mental Status Examination; RL/RI-16 = 16-item Free and Cued Recall; GDS = Geriatric Depression Scale; WHOQOL-BREF = World Health Organization Quality of Life-BREF; D1 = Physical health; D2 = Psychiatry; D3 = Social relationships; D4 = Environment * p < 0.05; ** p < 0.01; *** p < 0.001.

## Data Availability

Data are available from the corresponding author upon reasonable request.

## Supplementary Materials

The following supporting information can be downloaded at: https://www.mdpi.com/article/10.3390/ejihpe14020019/s1, Table S1: Results of the cognitive functions comparing three groups (CG, AT and ACT) at the four assessments (baseline, W4, W8 and W12); Table S2: Performances in different tasks of the three groups (CG, AT, and ACT) before and after activities (Reading, Pedaling, and Combined) and at 1-month follow-up.

## References

[1] The Epidemiology and Impact of Dementia: Current State and Future Trends. Genève, Organisation Mondiale de la Santé, 2015. Document WHO/MSD/MER/15.3. https://www.who.int/mental_health/neurology/dementia/dementia_thematicbrief_epidemiology.pdf.

[2] Ammar A., Boukhris O., Halfpaap N., Labott B.K., Langhans C., Herold F., Grässler B., Müller P., Trabelsi K., Chtourou H. (2021). Four Weeks of Detraining Induced by COVID-19 Reverse Cardiac Improvements from Eight Weeks of Fitness-Dance Training in Older Adults with Mild Cognitive Impairment. Int. J. Environ. Res. Public Health.

[3] Alzheimer’s Disease International World Alzheimer Report 2018: The State of the Art of Dementia Research: New Frontiers [EB/OL]. 1 September 2018. https://www.alz.co.uk/research/WorldAlzheimerReport2018.pdf.

[4] Petersen R.C., Smith G.E., Waring S.C., Ivnik R.J., Tangalos E.G., Kokmen E. (1999). Mild cognitive impairment: Clinical characterization and outcome. Arch. Neurol..

[5] Busse A., Bischkopf J., Riedel-Heller S.G., Angermeyer M.C. (2003). Mild cognitive impairment: Prevalence and incidence according to different diagnostic criteria. Results of the Leipzig Longitudinal Study of the Aged (LEILA75+). Br. J. Psychiatry.

[6] Barnes D.E., Yaffe K. (2011). The projected effect of risk factor reduction on Alzheimer’s disease prevalence. Lancet Neurol..

[7] Damirchi A., Hosseini F., Babaei P. (2018). Mental Training Enhances Cognitive Function and BDNF More than Either Physical or Combined Training in Elderly Women With MCI: A Small-Scale Study. Am. J. Alzheimers Dis. Other Demen..

[8] Northey J.M., Cherbuin N., Pumpa K.L., Smee D.J., Rattray B. (2017). Exercise interventions for cognitive function in adults older than 50: A systematic review with meta-analysis. Br. J. Sport. Med..

[9] Zheng G., Xia R., Zhou W., Tao J., Chen L. (2016). Aerobic exercise ameliorates cognitive function in older adults with mild cognitive impairment: A systematic review and meta-analysis of randomised controlled trials. Br. J. Sport. Med..

[10] Paumard C. (2014). Benefits of physical activity on chronic pathologies. Neurol. Psychiatr. Gériatrie.

[11] Cass S.P. (2017). Alzheimer’s Disease and Exercise: A Literature Review. Curr. Sport. Med. Rep..

[12] Haeger A., Costa A.S., Schulz J.B., Reetz K. (2019). Cerebral changes improved by physical activity during cognitive decline: A systematic review on MRI studies. Neuroimage Clin..

[13] Best J.R., Chiu B.K., Hall P.A., Liu-Ambrose T. (2017). Larger lateral prefrontal cortex volume predicts better exercise adherence among older women: Evidence from two exercise training studies. J. Gerontol. A Biol. Sci. Med. Sci..

[14] Ehlers D.K., Daugherty A.M., Burzynska A.Z., Fanning J., Awick E.A., Chaddock-Heyman L., Kramer A.F., McAuley E. (2017). Regional brain volumes moderate, but do not mediate, the effects of group-based exercise training on reductions in loneliness in older adults. Front. Aging Neurosci..

[15] Cammisuli D.M., Innocenti A., Fusi J., Franzoni F., Pruneti C. (2017). Aerobic exercise effect upon cognition in mild cognitive impairment: A systematic review of randomized controlled trials. Arch. Ital. Biol..

[16] Nuzum H., Stickel A., Corona M., Zeller M., Melrose R.J., Wilkins S.S. (2020). Potential Benefits of Physical Activity in MCI and Dementia. Behav. Neurol..

[17] Tricco A.C., Soobia C., Berliner S., Ho J.M., Ng C.H., Ashoor H.M., Chen M.H., Hemmelgarn B., Straus S.E. (2013). Efficacy and safety of cognitive enhancers for patients with mild cognitive impairment: A systematic review and meta-analysis. CMAJ.

[18] Russ T.C. (2014). Cholinesterase inhibitors should not be prescribed for mild cognitive impairment. Evid. Based Med..

[19] Zhou Y., Li L.D. (2022). Exercise training for cognitive and physical function in patients with mild cognitive impairment: A PRISMA-compliant systematic review and meta-analysis. Medicine.

[20] Herold F., Törpel A., Schega L., Schega L., Müller N.G. (2019). Functional and/or structural brain changes in response to resistance exercises and resistance training lead to cognitive improvements—A systematic review. Eur. Rev. Aging Phys. Act..

[21] Ströhle A., Schmidt D.K., Schultz F., Fricke N., Staden T., Hellweg R., Priller J., Rapp M.A., Rieckmann N. (2015). Drug and Exercise Treatment of Alzheimer Disease and Mild Cognitive Impairment: A Systematic Review and Meta-Analysis of Effects on Cognition in Randomized Controlled Trials. Am. J. Geriatr. Psychiatry.

[22] Sacco G., Caillaud C., Ben Sadoun G., Robert P., David R., Brisswalter J. (2016). Exercise Plus Cognitive Performance Over and Above Exercise Alone in Subjects with Mild Cognitive Impairment. J. Alzheimers Dis..

[23] Lampit A., Hallock H., Valenzuela M. (2014). Computerized cognitive training in cognitively healthy older adults: A systematic review and meta-analysis of effect modifiers. PLoS Med..

[24] Lampit A., Hallock H., Moss R., Kwok S., Rosser M., Lukjanenko M., Kohn A., Naismith S., Brodaty H., Valenzuela M. (2014). The time course of global cognitive gains from supervised computer assisted cognitive training: A randomised active controlled trail in elderly with multiple dementia risk factors. J. Prev. Alzheimers Dis..

[25] Zhu X., Yin S., Lang M., He R., Li J. (2016). The more the better? A metaanalysis on efects of combined cognitive and physical intervention on cognition in healthy older adults. Ageing Res. Rev..

[26] Karssemeijer E., Aaronson J.A., Bossers W.J., Smits T., Olde Rikkert M.G.M., Kessels R.P.C. (2017). Positive effects of combined cognitive and physical exercise training on cognitive function in older adults with mild cognitive impairment or dementia: A meta-analysis. Ageing Res. Rev..

[27] Schwenk M., Sabbagh M., Lin I., Morgan P., Grewal G.S., Mohler J., Coon D.W., Najafi B. (2016). Sensor-based balance training with motion feedback in people with mild cognitive impairment. J. Rehabil. Res. Dev..

[28] Delbroek T., Vermeylen W., Spildooren J. (2017). The efect of cognitive-motor dual task training with the biorescue force platform on cognition, balance and dual task performance in institutionalized older adults: A randomized controlled trial. J. Phys. Ther. Sci..

[29] Shimada H., Makizako H., Doi T., Park H., Tsutsumimoto K., Verghese J., Suzuki T. (2018). Effects of combined physical and cognitive exercises on cognition and mobility in patients with mild cognitive impairment: A randomized clinical trial. J. Am. Med. Dir. Assoc..

[30] Ben Ayed I., Castor-Guyonvarch N., Amimour S., Naija S., Aouichaoui C., Ben Omor S., Tabka Z., El Massioui F. (2021). Acute Exercise and Cognitive Function in Alzheimer’s Disease. J. Alzheimers Dis..

[31] Beck T.W. (2013). The importance of a priori sample size estimation in strength and conditioning research. J. Strength Cond. Res..

[32] Faul F., Erdfelder E., Lang A.G., Buchner A. (2007). G*Power 3: A flexible statistical power analysis program for the social, behavioral, and biomedical sciences. Behav. Res. Methods.

[33] Dubois B., Feldman H.H., Jacova C., Hampel H., Molinuevo J.L., Blennow K., DeKosk S.T., Gauthier S., Selkoe D., Bateman R. (2014). Advancing research diagnostic criteria for Alzheimer’s disease: The IWG-2 criteria. Lancet Neurol..

[34] Folstein M.F., Folstein S.E., McHugh P.R. (1975). “Mini-mental state”. A practical method for grading the cognitive state of patients for the clinician. J. Psychiatr. Res..

[35] Guyatt G.H., Sullivan M.J., Thompson P.J., Fallen E.L., Pugsley S.O., Taylor D.W., Berman L.B. (1985). The 6-minute walk: A new measure of exercise capacity in patients with chronic heart failure. Can. Med. Assoc. J..

[36] Brooks D., Solway S., Gibbons W.J. (2003). ATS statement on six-minute walk test. Am. J. Respir. Crit. Care Med..

[37] American Psychiatric Association (2013). Diagnostic and Statistical Manual of Mental Disorders: DSM-5.

[38] Dion M., Potvin O., Belleville S., Ferland G., Renaud M., Bherer L., Joubert S., Vallet G.T., Simard M., Rouleau I. (2015). Normative data for the Rappel libre/Rappel indicé à 16 items (16-item Free and Cued Recall) in the elderly Quebec-French population. Clin. Neuropsychol..

[39] Shulman K.I. (2000). Clock-drawing: Is it the ideal cognitive screening test?. Int. J. Geriatr. Psychiatry.

[40] Gomez R.G., White D.A. (2006). Using verbal fluency to detect very mild dementia of the Alzheimer type. Arch. Clin. Neuropsychol..

[41] Brown P.J., Woods C.M., Storandt M. (2007). Model stability of the 15-item Geriatric Depression Scale across cognitive impairment and severe depression. Psychol. Aging.

[42] Lucas-Carrasco R., Skevington S.M., Gómez-Benito J., Rejas J., March J. (2011). Using the WHOQOL-BREF in persons with dementia: A validation study. Alzheimer Dis. Assoc. Disord..

[43] Troyer A.K., Leach L., Strauss E. (2007). Aging and Response Inhibition: Normative Data for the Victoria Stroop Test. Aging Neuropsychol. Cogn..

[44] Scarpina F., Tagini S. (2017). The Stroop Color and Word Test. Front. Psychol..

[45] Gregoire J., Van der Linden M. (1997). Effect of age on forward and backward digit spans. Aging Neuropsychol. Cogn..

[46] Monaco M., Costa A., Caltagirone C., Carlesimo G.A. (2013). Forward and backward span for verbal and visuo-spatial data: Standardization and normative data from an Italian adult population. Neurol. Sci..

[47] Muangpaisan W., Intalapaporn S., Assantachai. P. (2010). Digit span and verbal fluency tests in patients with mild cognitive impairment and normal subjects in Thai-community. J. Med. Assoc. Thail..

[48] Anderson J., Douglass S. (2001). Tower of Hanoi: Evidence for the cost of goal retrieval. J. Exp. Psychol. Learn. Mem. Cogn..

[49] de Oliveira Silva F., Ferreira J.V., Plácido J., Sant’Anna P., Araújo J., Marinho V., Laks J., Deslandes A.C. (2019). Three months of multimodal training contributes to mobility and executive function in elderly individuals with mild cognitive impairment, but not in those with Alzheimer’s disease: A randomized controlled trial. Maturitas.

[50] Fonte C., Smania N., Pedrinolla A., Munari D., Gandolfi M., Picelli A., Varalta V., Benetti M.V., Brugnera A., Federico A. (2019). Comparison between physical and cognitive treatment in patients with MIC and Alzheimer’s disease. Aging.

[51] Park H., Park J.H., Na H.R., Hiroyuki S., Kim G.M., Jung M.K., Kim W.K., Park K.W. (2019). Combined Intervention of Physical Activity, Aerobic Exercise, and Cognitive Exercise Intervention to Prevent Cognitive Decline for Patients with Mild Cognitive Impairment: A Randomized Controlled Clinical Study. J. Clin. Med..

[52] tenBrinke L.F., Bolandzadeh N., Nagamatsu L.S., Hsu C.L., Davis J.C., Miran-Khan K., Liu-Ambrose T. (2015). Aerobic exercise increases hippocampal volume in older women with probable mild cognitive impairment: A 6-month randomised controlled trial. Br. J. Sport. Med..

[53] Meng Q., Yin H., Wang S., Shang B., Meng X., Yan M., Li G., Chu J., Chen L. (2022). The effect of combined cognitive intervention and physical exercise on cognitive function in older adults with mild cognitive impairment: A meta-analysis of randomized controlled trials. Aging Clin. Exp. Res..

[54] Gavelin H.M., Dong C., Minkov R., Bahar-Fuchs A., Ellis K.A., Lautenschlager N.T., Mellow M.L., Wade A.T., Smith A.E., Finke C. (2021). Combined physical and cognitive training for older adults with and without cognitive impairment: A systematic review and network meta-analysis of randomized controlled trials. Ageing Res. Rev..

[55] Salzman T., Sarquis-Adamson Y., Son S., Montero-Odasso M., Fraser S. (2022). Associations of Multidomain Interventions With Improvements in Cognition in Mild Cognitive Impairment: A Systematic Review and Meta-analysis. JAMA Netw. Open.

[56] Xue D., Li P.W.C., Yu D.S.F., Rose S.Y., Lin R.S.Y. (2023). Combined exercise and cognitive interventions for adults with mild cognitive impairment and dementia: A systematic review and network meta-analysis. Int. J. Nurs. Stud..

[57] Uysal I., Başar S., Aysel S., Kalafat D., Büyüksünnetçi A.Ö. (2023). Aerobic exercise and dual-task training combination is the best combination for improving cognitive status, mobility and physical performance in older adults with mild cognitive impairment. Aging Clin. Exp. Res..

[58] Anderson-Hanley C., Barcelos N.M., Zimmerman E.A., Gillen R.W., Dunnam M., Cohen B.D., Yerokhin V., Miller K.E., Hayes D.J., Arciero P.J. (2018). The aerobic and cognitive exercise study (ACES) for community-dwelling older adults with or at-risk for mild cognitive impairment (MCI): Neuropsychological, neurobiological and neuroimaging outcomes of a randomized clinical trial. Front. Aging Neurosci..

[59] Beaunieux H., Eustache F., Busson P., de la Sayette V., Viader F., Desgranges B. (2012). Cognitive procedural learning in early Alzheimer’s disease: Impaired processes and compensatory mechanisms. J. Neuropsychol..

[60] Chirles T.J., Reiter K., Weiss L.R., Alfini A.J., Nielson K.A., Smith J.C. (2017). Exercise Training and Functional Connectivity Changes in Mild Cognitive Impairment and Healthy Elders. J. Alzheimers Dis..

[61] Meeusen R. (2014). Exercise, nutrition and the brain. Sport. Med..

[62] Firth J., Stubbs B., Vancampfort D., Schuch F., Lagopoulos J., Rosenbaum S., Ward P.B. (2018). Effect of aerobic exercise on hippocampal volume in humans: A systematic review and meta-analysis. Neuroimage.

[63] Van Balkom T.D., van den Heuvel O.A., Berendse H.W., van der Werf Y.D., Vriend C. (2020). The Effects of Cognitive Training on Brain Network Activity and Connectivity in Aging and Neurodegenerative Diseases: A Systematic Review. Neuropsychol. Rev..

[64] Stigger F.S., Zago Marcolino M.A., Portela K.M., Portela K.M., Méa Plentz R.D. (2019). Effects of Exercise on Inflammatory, Oxidative, and Neurotrophic Biomarkers on Cognitively Impaired Individuals Diagnosed with Dementia or Mild Cognitive Impairment: A Systematic Review and Meta-Analysis. J. Gerontol. A Biol. Sci. Med. Sci..

[65] Vazquez-Sanroman D., Sanchis-Segura C., Toledo R., Hernandez M.E., Manzo J., Miquel M. (2013). The effects of enriched environment on 8 American Journal of Alzheimer’s Disease & Other Dementias^®^ XX(X) BDNF expression in the mouse cerebellum depending on the length of exposure. Behav. Brain Res..

[66] Pressler S., Titler M., Koelling T.M., Riley P.L., Miyeon Jung M., Hoyland-Domenico L., Ronis D.L., Smith D.G., Bleske B.E., Dorsey S.G. (2015). Nurse-enhanced computerized cognitive training increases serum brain derived neurotrophic factor levels and improves working memory in heart failure. J. Card. Fail..

[67] Byun J.E., Kang E.B. (2016). The effects of senior brain health exercise program on basic physical fitness, cognitive function and BDNF of elderly women a feasibility study. J. Exerc. Nutr. Biochem..

[68] Nascimento C.M.C., Pereira J.R., Pires de Andrade L., Garuffi M., Ayan C., Shikanai K.S., Talib L.L., Cominetti M.R., Stella F. (2015). Physical exercise improves peripheral BDNF levels and cognitive functions in mild cognitive impairment elderly with different BDNF Val66Met genotypes. J. Alzheimers Dis..

[69] Håkansson K., Ledreux A., Daffner K., Terjestam Y., Bergman P., Carlsson R., Kivipelto M., Winblad B., Granholm A.C.H., Mohammed A.K. (2017). BDNF Responses in Healthy Older Persons to 35 Minutes of Physical Exercise, Cognitive Training, and Mindfulness: Associations with Working Memory Function. J. Alzheimers Dis..

[70] Ide K., Secher N.H. (2000). Cerebral blood flow and metabolism during exercise. Prog. Neurobiol..

[71] Zimmerman B., Sutton B.P., Low K.A., Fletcher M.A., Hong Tan C., Schneider-Garce N., Li Y., Ouyang C.L., Maclin E., Gratton G. (2014). Cardiorespiratory fitness mediates the effects of aging on cerebral blood flow. Front. Aging Neurosci..

[72] Binnewijzend M.A.A., Kuijer J.P.A., Benedictus M.R., van der Flier W.M., Meije Wink A., Wattjes M.P., van Berckel B.N.M., Scheltens P., Barkhof F. (2013). Cerebral blood flow measured with 3D pseudocontinuous arterial spin-labeling MR imaging in Alzheimer disease and mild cognitive impairment: A marker for disease severity. Radiology.

[73] Lambourne K., Audiffren M., Tomporowski P.D. (2010). Effects of acute exercise on sensory and executive processing tasks. Med. Sci. Sport. Exerc..

[74] Wang Y.-Y., Wang X.-X., Chen L., Liu Y., Li Y.-R. (2023). A systematic review and network meta-analysis comparing various non-pharmacological treatments for older people with mild cognitive impairment. Asian J. Psychiatr..

