# Supplementary material for: Mid-Term and Long-Lasting Psycho–Cognitive Benefits of Bidomain Training Intervention in Elderly Individuals with Mild Cognitive Impairment
Source: Eur J Investig Health Psychol Educ. 2024 Jan 26;14(2):284–98. doi: 10.3390/ejihpe14020019 (PMC10887966; doi:10.3390/ejihpe14020019)
Supplement: Supplementary file 1 [file ejihpe-14-00019-s001.zip › ejihpe-2784325-supplementary.pdf]

Supplementary file

Table S1. Results of the cognitive functions comparing three groups (CG, AT and ACT) at the four assessments (baseline, W4, W8 and W12)

Groups<br>Tasks	Control group (n = 14)	Aerobic Training (n = 15)	Aerobic-Cognitive Training (n = 15)	Kruskal–Wallis Analysis	Post Hoc Comparison AT vs. ACT
	M (SD)	M (SD)	M (SD)	H	H
**Cognitive Functions Baseline**					
Stroop interference score	7.85 (0.77)	7.60 (0.80)	7.20 (0.83)	4.21	4.21
Hanoi execution time	75.07(8.57)	74.86(10.51)	72.73 (8.42)	1.15	1.15
Hanoi number of moves	10.50(3.63)	10.79 (3.49)	10.26 (3.49)	0.13	0.13
Digit Span Forward	3.47 (0.21)	3.52 (0.14)	3.50 (0.09)	0.56	0.56
Digit Span backward	3.28 (0.18)	3.30 (0.16)	3.37 (0.16)	2.05	2.05
**Cognitive Functions after 4 weeks (W4)**					
Stroop interference score	8.21 (0.69)	6.13 (0.74)	5.86 (0.91)	26.21***	26.21
Hanoi execution time	75.28(8.31)	63.46 (9.57)	58.46 (10.50)	14.60***	14.60
Hanoi number of moves	11 (3.59)	9.80 (3.54)	8.40 (2.89)	4.14	4.14
Digit Span Forward	3.44 (0.17)	3.61 (0.14)	3.77 (0.07)	24.15***	24.15
Digit Span backward	3.27 (0.18)	3.44 (0.08)	3.61 (0.09)	25.06***	25.06**
**Cognitive Functions after 8 weeks (W8)**					
Stroop interference score	8.78 (1.52)	5.33 (0.61)	4.33 (0.48)	32.46***	32.46**
Hanoi execution time	81.35(7.55)	51.53 (8.02)	42.46 (8.25)	31.01***	31.01*
Hanoi number of moves	11.50(3.48)	7.93 (2.46)	7.00 (0.00)	17.21***	17.21*
Digit Span Forward	3.38 (0.16)	3.92 (0.10)	4.00 (0.00)	35.74***	35.74*
Digit Span backward	3.30 (0.23)	3.62 (0.10)	3.78 (0.14)	27.96***	27.96**
**Cognitive Functions after 4-week of rest (W12)**					
Stroop interference score	9.21 (0.97)	5.26 (0.79)	4.26 (0.45)	34.10***	34.10**
Hanoi execution time	81.07(8.64)	50.13 (6.85)	42.93 (8.38)	30.51***	30.51
Hanoi number of moves	11.00(3.59)	7.46 (1.80)	7.00 (0.00)	16.80***	16.80
Digit Span Forward	3.34 (0.18)	3.90 (0.10)	3.98 (0.05)	34.22***	34.22
Digit Span backward	3.24 (0.16)	3.64 (0.15)	3.80 (0.13)	31.07***	31.07

M = mean; SD = standard deviation; * $p < 0.05$; ** $p < 0.01$; ***$p < 0.001$.

Table S2. Performances in different tasks of the three groups (CG, AT, and ACT) before and after activities (Reading, Pedaling, and Combined) and follow-up 1 month

Training duration	Baseline	W4	W8	Friedman analysis	W12	W8vsW12
**Groups**	**M (SD)**	**M (SD)**	**M (SD)**	**Khi²**	**M (SD)**	**z**
**Control group (n = 14)**						
**Stroop interference score**	7.85 (0.77)	8.21 (0.69)	8.78 (1.52)	6.95	9.21 (0.97)	-0.97
**Hanoi execution time**	75.07 (8.57)	75.28 (8.31)	81.35 (7.55)	7.42	81.07 (8.64)	-0.36
**Hanoi number of moves**	10.50 (3.63)	11 (3.59)	11.5 (3.48)	1.20	11.00 (3.59)	-1.00
**Digit Span Forward**	3.47 (0.21)	3.44 (0.17)	3.38 (0.16)	1.55	3.34 (0.18)	-0.78
**Digit Span backward**	3.28 (0.18)	3.27 (0.18)	3.30 (0.23)	0.30	3.24 (0.16)	-0.73
**Aerobic Training group (n= 15)**						
**Stroop interference score**	7.60 (0.80)	6.13 (0.74)	5.33 (0.61)	26.53***	5.26 (0.79)	-0.28
**Hanoi execution time**	74.86(10.51)	63.46 (9.57)	51.53 (8.02)	25.12***	50.13 (6.85)	-1.66
**Hanoi number of moves**	10.79 (3.49)	9.8 (3.54)	7.93 (2.46)	5.60**	7.46 (1.80)	-1.00
**Digit Span Forward**	3.52 (0.14)	3.61 (0.14)	3.92 (0.10)	23.81***	3.90 (0.10)	-0.38
**Digit Span backward**	3.30 (0.16)	3.44 (0.08)	3.62 (0.10)	22.33***	3.64 (0.15)	-0.45
**Aerobic-Cognitive Training (n=15)**						
**Stroop interference score**	7.20 (0.83)	5.86 (0.91)	4.33 (0.48)	26.56***	4.26 (0.45)	-1.00
**Hanoi execution time**	72.73 (8.42)	58.46 (10.50)	42.46 (8.25)	30.00***	42.93 (8.38)	-0.51
**Hanoi number of moves**	10.26 (3.49)	8.4 (2.89)	7.00 (0.00)	9.25**	7.00 (0.00)	-1.00
**Digit Span Forward**	3.50 (0.09)	3.77 (0.07)	4.00 (0.00)	30.00***	3.98 (0.05)	-1.00
**Digit Span backward**	3.37 (0.16)	3.61 (0.09)	3.78 (0.14)	25.32***	3.80 (0.13)	-0.38

M = mean; SD = standard deviation; **** $p < 0.05$; ** $p < 0.01$; ***$p < 0.001$***
